# The Challenge of Regulation in a Minimal Photoautotroph: Non-Coding RNAs in *Prochlorococcus*

**DOI:** 10.1371/journal.pgen.1000173

**Published:** 2008-08-29

**Authors:** Claudia Steglich, Matthias E. Futschik, Debbie Lindell, Bjoern Voss, Sallie W. Chisholm, Wolfgang R. Hess

**Affiliations:** 1 Faculty of Biology, University of Freiburg, Freiburg, Germany; 2 Institute of Theoretical Biology, Humboldt University, Berlin, Germany; 3 Faculty of Biology, Technion – Israel Institute of Technology, Haifa, Israel; 4 Department of Civil and Environmental Engineering, Massachusetts Institute of Technology, Cambridge, Massachusetts, United States of America; Université Paris Descartes, INSERM U571, France

## Abstract

*Prochlorococcus*, an extremely small cyanobacterium that is very abundant in the world's oceans, has a very streamlined genome. On average, these cells have about 2,000 genes and very few regulatory proteins. The limited capability of regulation is thought to be a result of selection imposed by a relatively stable environment in combination with a very small genome. Furthermore, only ten non-coding RNAs (ncRNAs), which play crucial regulatory roles in all forms of life, have been described in *Prochlorococcus*. Most strains also lack the RNA chaperone Hfq, raising the question of how important this mode of regulation is for these cells. To explore this question, we examined the transcription of intergenic regions of *Prochlorococcus* MED4 cells subjected to a number of different stress conditions: changes in light qualities and quantities, phage infection, or phosphorus starvation. Analysis of Affymetrix microarray expression data from intergenic regions revealed 276 novel transcriptional units. Among these were 12 new ncRNAs, 24 antisense RNAs (asRNAs), as well as 113 short mRNAs. Two additional ncRNAs were identified by homology, and all 14 new ncRNAs were independently verified by Northern hybridization and 5′RACE. Unlike its reduced suite of regulatory proteins, the number of ncRNAs relative to genome size in *Prochlorococcus* is comparable to that found in other bacteria, suggesting that RNA regulators likely play a major role in regulation in this group. Moreover, the ncRNAs are concentrated in previously identified genomic islands, which carry genes of significance to the ecology of this organism, many of which are not of cyanobacterial origin. Expression profiles of some of these ncRNAs suggest involvement in light stress adaptation and/or the response to phage infection consistent with their location in the hypervariable genomic islands.

## Author Summary

*Prochlorococcus* is the most abundant phototroph in the vast, nutrient-poor areas of the ocean. It plays an important role in the ocean carbon cycle, and is a key component of the base of the food web. All cells share a core set of about 1,200 genes, augmented with a variable number of “flexible” genes. Many of the latter are located in genomic islands—hypervariable regions of the genome that encode functions important in differentiating the niches of “ecotypes.” Of major interest is how cells with such a small genome regulate cellular processes, as they lack many of the regulatory proteins commonly found in bacteria. We show here that contrary to the regulatory proteins, ncRNAs are present at levels typical of bacteria, revealing that they might have a disproportional regulatory role in *Prochlorococcus*—likely an adaptation to the extremely low-nutrient conditions of the open oceans, combined with the constraints of a small genome. Some of the ncRNAs were differentially expressed under stress conditions, and a high number of them were found to be associated with genomic islands, suggesting functional links between these RNAs and the response of *Prochlorococcus* to particular environmental challenges.

## Introduction

Cyanobacteria are a diverse group of photoautotrophic bacteria that occupy a broad range of habitats, including the oceans, lakes, and soil, and are also found as symbionts in many different types of organisms. *Prochlorococcus*, a member of the cyanobacterial lineage, often accounts for up to 50% of the photosynthetic biomass in the open oceans between 40°N and 40°S [1],[2]. In these areas *Prochlorococcus* numerically dominates the phytoplankton with cell numbers reaching 10^5^ cells per mL [3]. Two major ecotypes can be differentiated within the *Prochlorococcus* group, which are relatively adapted to high or low light. They are genetically and physiologically distinct [4] and are distributed differently in the water column [5]–[7], with the high light adapted cells dominating the surface waters, and the low light adapted cells abundant in deep waters.

The genomes of 12 *Prochlorococcus* strains, spanning the known microdiversity within the group, have been sequenced (http://www.ncbi.nlm.nih.gov/genomes/MICROBES/microbial_taxtree.html). The cells posses the most streamlined genome of a free-living photoautotroph with genome sizes ranging from 1.6 Mbp to 2.7 Mbp [8]–[10]. The number of modelled protein-coding genes in these genomes is 1,855–3,022 [9] and the core genome shared by all *Prochlorococcus* strains has been estimated at 1,273 genes [9]. Several hundred additional genes are specific for one or only a few strains, and they are frequently clustered in genomic islands [11],[9].

Genome reduction in this genus has particularly affected the number of regulatory genes. Many otherwise widely distributed two-component systems and DNA-binding proteins are not present in *Prochlorococcus*. This has been linked to the fitness gain conferred by a streamlined genome to organisms existing in a relatively stable environment [8]. Although the ocean environment may be relatively stable, it does fluctuate, making one wonder how *Prochlorococcus* cells respond to these changing conditions. Perhaps each protein regulator performs multiple regulatory functions in this cell. Alternatively, non-coding RNAs (ncRNAs) may play a major regulatory role compensating the lack of regulatory proteins.

ncRNAs are functional RNA molecules, mostly without a protein-coding function, and their genes are normally located in intergenic regions. They frequently play a crucial role in bacterial regulatory networks particularly in response to environmental stress [12],[13] and are also known to control plasmid and viral replication [14], bacterial virulence [15] and quorum sensing [16]. However the function of many ncRNAs remains unknown. *Escherichia coli* has over 70 ncRNAs most of which have been detected by computational prediction [17]–[20] and “experimental RNomics” [21]–[23]. These regulators were overlooked by traditional genome annotation due to their short length (50–400 nt in size), the lack of algorithms to search for sequences that are frequently more conserved in secondary structure rather than sequence, and the absence of a protein coding function.

Another class of functional RNAs – chromosomally encoded antisense RNAs (asRNAs) also plays a role in the regulation of gene expression. There are no systematic approaches to screen for asRNAs, but RNomics approaches have inadvertently revealed the presence of asRNAs in *Escherichia coli* [21]–[23]. These *cis*-encoded asRNAs are transcribed from the opposite strand of the same genomic locus as the target (m)RNA and feature 100% base complementarity. In contrast, most ncRNAs studied so far act in *trans* in a different genomic locus having only a short and imperfect base complementarity with the target transcripts (for a detailed review see [24]).

Although a considerable number of *Prochlorococcus* strains have been fully sequenced, only a small number of ncRNAs have been identified in this group. In addition to the ubiquitous signal recognition particle RNA, RNAse P RNA and the tmRNA, encoded by *ffs*, *rnpB* and *ssrA*, seven ncRNAs have been identified in cyanobacteria, all of which were first described in *Prochlorococcus* MED4 and were denoted as Yfr1–Yfr7, for cYanobacterial Functional RNA [25]. Amongst them is Yfr7, which is homologous to 6S RNA [26] and known to have global regulatory functions in *Escherichia coli*. Another is Yfr1, which has homologues in other cyanobacteria [27] and in *Synechococcus elongatus* PCC6301, is required for growth under multiple stress conditions [28]. These two ncRNAs were classified as such after experimental verification of the expression of candidate ncRNAs initially identified from secondary structure conservation using a comparative genomics approach. Little is known regarding *cis*-acting asRNAs in cyanobacteria. Only 3 chromosomally *cis*-encoded asRNAs have been identified so far [29]–[31], none of which occur in *Prochlorococcus*.

Despite the presence of ncRNAs in *Prochlorococcus* the gene encoding the Hfq RNA chaperone is absent from 10 of the 12 sequenced *Prochlorococcus* strains, including MED4. This is in contrast to other completely sequenced cyanobacteria that all contain an Hfq homologue. Hfq belongs to the eukaryotic and archaeal family of Sm and Sm-like (Lsm) proteins and is found in all domains of life. It facilitates the interaction of ncRNAs with their target mRNAs and is thus involved in many essential regulatory processes including ncRNA-mediated translational regulation [32]–[35]. Its loss during evolution of the *Prochlorococcus* group may be taken as evidence for a general decay in RNA-dependent gene regulation or as an indication that novel mechanisms for RNA - RNA interactions may exist in this group.

In the past few years, new experimental strategies such as ‘experimental RNomics’ and mining microarray expression data in intergenic regions have demonstrated that the number of ncRNAs in microbial genomes is much greater than previously thought (for reviews see [36],[37]). In the light of the small number of ncRNAs detected thus far in *Prochlorococcus* we were curious to see whether more ncRNAs are present in *Prochlorococcus* than were detected in the comparative genomics analysis used by Axman et al. [25].

Using an alternative approach based on microarray expression profiling, we investigated the presence of ncRNAs in *Prochlorococcus* MED4, which has the most compact genome of all sequenced *Prochlorococcus* strains, has few protein coding regulators, and is Hfq-deficient.

## Results/Discussion

### Identification of Novel Transcripts

**Figure 1:**
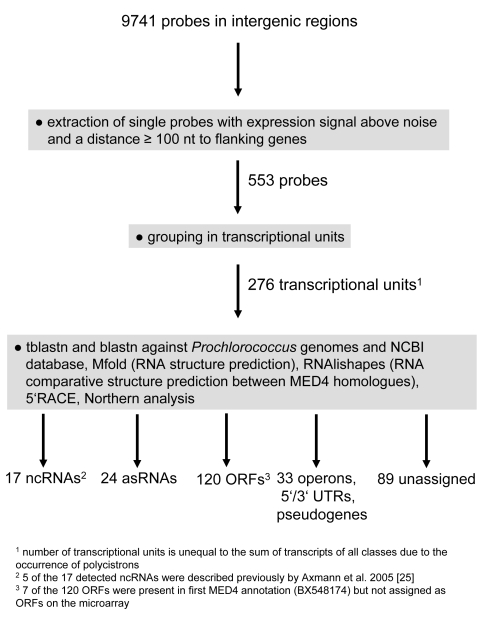
Computational pipeline for the identification of novel genetic elements from microarray data. The data were derived from experiments studying stress of *Prochlorococcus* MED4 induced by light changes, phosphorus starvation, and phage infection. Note that the sum of classified transcripts is unequal to the number of transcriptional units due to the occurrence of dicistronic elements. In addition to the 12 ncRNAs newly detected by microarray analysis, two ncRNAs Yfr12 and Yfr18 were found based on similarity.

The design of the *Prochlorococcus* custom Affymetrix microarray, which contains probes not only in gene-coding regions but also in intergenic regions (on both strands), and the availability of diverse data sets describing changes in gene expression in response to environmental stresses, allowed us to undertake a focused study of transcriptionally active intergenic regions. Three independent data sets were used: experiments investigating global changes in gene expression under different light quantities and qualities (from here on referred to as the “light experiment”, [38]), under phage infection (the “phage experiment”, [39]), and under phosphorus starvation (the “phosphorus experiment”, [40]), encompassing a total of 95 microarrays. To designate expression signals as novel transcripts, probes had to be above a threshold expression level, and be further than 100 nt from flanking genes (see Materials and Methods for details). After identifying 553 probes that met these criteria from the light, phage and phosphorus experiments, we combined adjacent probes, yielding 276 unique transcriptional units (Figure 1). These transcripts were classified as 5′-UTRs, 3′–UTRs, operon elements, pseudogenes and “other” transcripts based on their genome location and experimental information. The “other” transcripts were then classified: as i) ORFs if they had a protein-coding reading frame with a start and stop codon without a frame shift in *Prochlorococcus* MED4 and in the genome of at least one other *Prochlorococcus* strain; ii) ncRNAs if they lacked an ORF, but had structural features typical of ncRNAs, such as compensatory mutations; and iii) asRNAs if they were located on the opposite strand of mRNAs. No assignment was made for 89 transcripts which either could not be verified in independent experiments (see below) or did not have homologues in other genomes. While some of these unclassified transcripts may represent unverified small ORFs or ncRNAs, they may also be the result of artificial expression signals. These may have occurred due to: i) cross-hybridization with duplicated regions that have only small sequence differences; or ii) artificial antisense signals caused by self-priming through hairpin loop extension of the first-strand cDNA, re-priming either from RNA fragments formed during degradation of the RNA templates, or from primers present in the reaction [41].

### Identification of New ncRNAs

**Figure 2:**
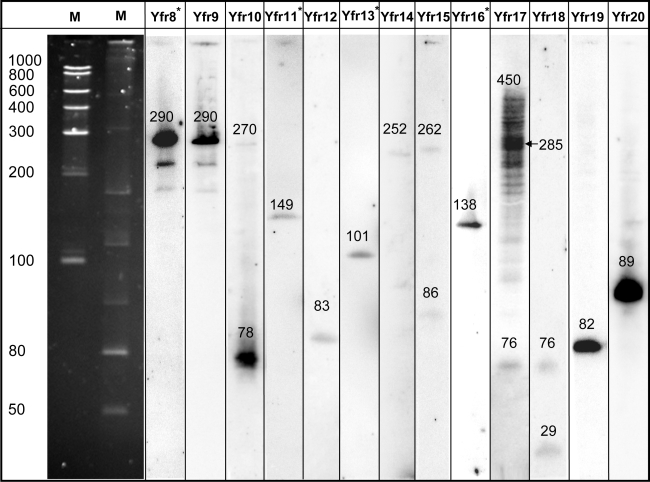
Detection of novel ncRNAs by Northern hybridization. The lengths of hybridizing fragments were calculated by comparison to two different size markers (M) – size in nt is shown to the left of the markers. 50 µg of total RNA was loaded per lane on a 10% polyacrylamide gel that was electro-blotted on Hybond-N nylon membranes. All ncRNAs were detected with single strand RNA probes except for those labeled with an asterisk, which were hybridized to an oligonucleotide probe.

Twelve novel ncRNAs were identified through microarray analyses. They were verified in independent experiments by rapid analysis of cDNA 5′ ends (5′ RACEs) and Northern hybridizations, which also served to map their first nucleotide and estimate their lengths (Table 1, Figure 2). In addition, 5 of the 7 previously described ncRNAs (Yfr2 and Yfr4–Yfr7, [25]) were also detected. Yfr1 may not have been detected due to its extraordinary small size (54 nt) which may have resulted in its removal during the cDNA clean-up process. Regardless, Yfr1 would be excluded from our analysis because of its close proximity to an annotated protein-coding *trxA* gene (within 100 nt) that is transcribed in the same direction. Expression levels of Yfr3 were the lowest of the previously reported ncRNAs [25], which likely explains why we did not detect this ncRNA in the microarray analysis. The internal consistency of these findings provide confidence in our approach, and suggest that even more ncRNAs may exist in *Prochlorococcus*, especially if they are very short, minimally expressed or close to protein-coding genes. Indeed two additional ncRNAs (Yfr12 and Yfr18) are described below that were identified by sequence homology, and that were not found through the microarray analysis because their expression signal was below the set threshold value.

**Table 1:** Synopsis of ncRNAs in *Prochlorococcus* MED4. Newly identified ncRNAs are based on microarray expression data or on sequence similarity (Yfr12 and Yfr18) according to the scheme shown in Figure 1.

probe names	new name	genomic location	length in nt^a^	adjacent genes	genomic context	occurence in *Prochlorococcus*	Genomic island	mean MFE z score^b^	SVM RNA-class probability^b^
IG0367f11-17	Yfr8	339952..340241	180, 220, 290	PMED4_03781 and PMED4_03791 (PMM0356)	→ → →	MIT9301, MIT9515, MIT9312, AS9601, MIT9215	1	−4.64	0.999616
IG0367r2-5	Yfr9	complement(339887..340176)	180, 220, 290	PMED4_03781 and PMED4_03791 (PMM0356)	→ ← →	MIT9301, MIT9515, MIT9312, AS9601, MIT9215	1	−2.54	0.676448
IG0375f18-20	Yfr10	346661..346738	78, 270^e^	PMED4_03891 and Yfr2	← → →	MIT9301, MIT9312	1	−4.69^j^	0.991539^j^
IG0390r20-22	Yfr11	complement(358606..358754)	149	PMED4_04111 and PMED4_04121					
(PMM0378)	← ← ←	MIT9515	1	−2.38^f^	0.998347^f^				
-	Yfr12	383230..383312	83	PMED4_04471 and Yfr4	→ → →	MIT9301, MIT9312	-	−3.09^j^	0.996117^j^
IG0668r5	Yfr13	complement(621601..621701)	101	PMED4_07021 and PMED4_07031					
(PMM0652)	← ← ←	MIT9301, MIT9515, MIT9312, MIT9215, NATL1a, NATL2a	-	−1.78^g^	0.600913^g^				
IG0676f22-24	Yfr14	627811..628062	252	PMED4_07142 and PMED4_07151	→ → →	n.d.	-	n.d.	n.d.
IG0702r18-21	Yfr15	complement(654301..654562)	86, 262	PMED4_07441 and Yfr3	→ ← →	n.d.	2	n.d.	n.d.
IG0704r24-25	Yfr16	complement(657392..657529)	138	PMED4_07521 and PMED4_07522	← ← →	MIT9515	2	−2.38^f^	0.998347^f^
IG0714f13	Yfr17	664667..664930^d^	76, 285, 450	PMED4_07691 and PMED4_07701 (PMM0699)	← → →	MIT9301, MIT9515, MIT9312,AS9601, MIT9215	-	−1.05^h^	0.000000^h^
-	Yfr18	complement(972284..972388)	29, 76	Yfr5 and PMED4_11681	← ← →	MIT9301, MIT9515, MIT9312, AS9601, MIT9215	-	−0.92^i^	0.996639^i^
IG1142r19-25	Yfr21^c^	complement(1069553..1069757)	205	PMED4_12671 and PMED4_12681					
(PMM1121)	← ← ←	no homologues	-	n.d.	n.d.				
IG1157r8	Yfr19	complement(1086081..1086162)	82	PMED4_12891 and PMED4_12901					
(PMM1135)	→ ← →	MIT9301, MIT9312, AS9601, MIT9215	-	−3.89^g^	0.999504^g^				
IG1412f30	Yfr20	1336435..1336523	89	PMED4_15791 and PMED4_15792	→ → →	MIT9515, MIT9312	5	−0.77	0.142982

Microarray probe IDs are given together with the respective ncRNA name, location and length. Computational support values (mean MFE z score and SVM RNA class probability) have been calculated according to Washietl et al. [65].

a length determined from Northern results and verified by 5′RACE.

b values derived from RNAz.

c not detected by Northern blot.

d length and location determined from 5′RACE results.

e precursor of Yfr10 and Yfr2.

f values derived from consensus structure of Yfr11, Yfr16 and a single homologue in MIT9515.

g values without MIT9301, MIT9312 and MIT9215.

h values derived from 264 nt long RNA as determined by 5′RACE; without AS9601, MIT9301 and MIT9215.

i values without MIT9301 and MIT9215.

j values derived using the same MIT9301 and MIT9312 homologues.

### Distribution of ncRNAs within the MED4 Genome and Those of Other *Prochlorococcus* Strains

**Figure 3:**
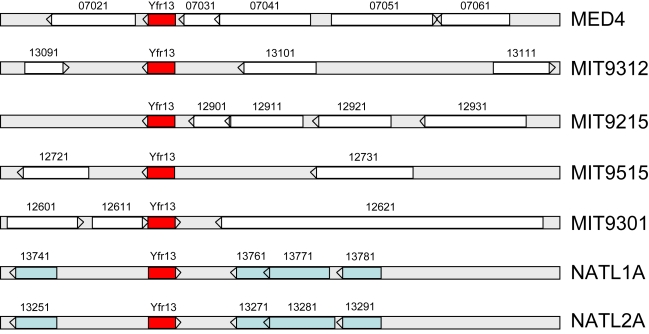
Genomic location of Yfr13 (red boxes) in seven different *Prochlorococcus* strains. Homologous genes in different strains are indicated by blue boxes and those without homology in this genomic region are shown in white. Gene designations are given according to published genome sequences for MED4 (BX548174.1), MIT9312 (CP000111.1), MIT9515 (CP000552.1), MIT9215 (CP000825), MIT9301 (CP000576.1), NATL1A (CP000553.1), NATL2A (CP000095.2).

Unlike Yfr1, which a sequence motif-based approach [27] revealed has homologues throughout the cyanobacterial lineage, none of our newly detected ncRNAs were universally present among the cyanobacteria. Indeed BlastN analyses yielded no evidence for their existence outside of the *Prochlorococcus* genus. With the exception of Yfr13, homologues of newly identified ncRNAs were only found in other high light-adapted *Prochlorococcus* strains (Table 1). Yfr13 has homologues in seven different *Prochlorococcus* strains, including the two low light-adapted isolates NATL1A and NATL2A, although the genome location is variable in the different strains (Figure 3).

ncRNAs are non-randomly distributed within the MED4 genome. They are often associated with hypervariable genomic islands, thought to arise by horizontal gene transfer [11]. MED4 has 5 genomic islands that constitute only about one tenth of the total genome, whereas 9 of the 21 ncRNAs, described here or by Axmann et al. [25], are in one of these islands (Table 1). The majority of island-associated ncRNAs are located in island 1 (Yfr8–Yfr11 and Yfr2). Three additional ncRNAs occur in island 2 (Yfr15, Yfr16 and Yfr3) and one is found in island 5 (Yfr20). Interestingly, the homologous ncRNAs in other *Prochlorococcus* strains are not always located in the corresponding island but occur somewhere else in the genome. The reverse is also true: some ncRNAs that are not island-associated in MED4 are located in an island region in other strains, indicative of recombination events. No ncRNAs were detected in island 4 even though this is the largest (74.5 kb long) of all islands present in MED4. Island 4 mainly encodes cell surface-relevant proteins such as glycosyltransferases or lipopolysaccharide-forming enzymes [11], suggesting these functions are not controlled through ncRNAs. Yfr11 and Yfr16 are highly similar to each other. Based on their sequence identity (74%) and their highly similar secondary structures (Figure S1) both ncRNAs may regulate the same targets as has been shown for PrrF1 and PrrF2 in *Pseudomonas aeruginosa* [42] and for Qrr1, Qrr2, Qrr3, and Qrr4 in several *Vibrio* species [16]. Alternatively, they might act in a related context but with non-identical functions as has recently been described for GlmY and GlmZ ncRNAs of *Escherichia coli* [43]. Alternatively, Yfr11 and Yfr16 could be functionally equivalent but expressed in a different regulatory context, as is frequently the case for protein-coding genes that occur in multiple copies in a single genome.

### Differential Expression Suggests the Involvement of ncRNAs in Regulation during Stress Conditions

**Figure 4:**
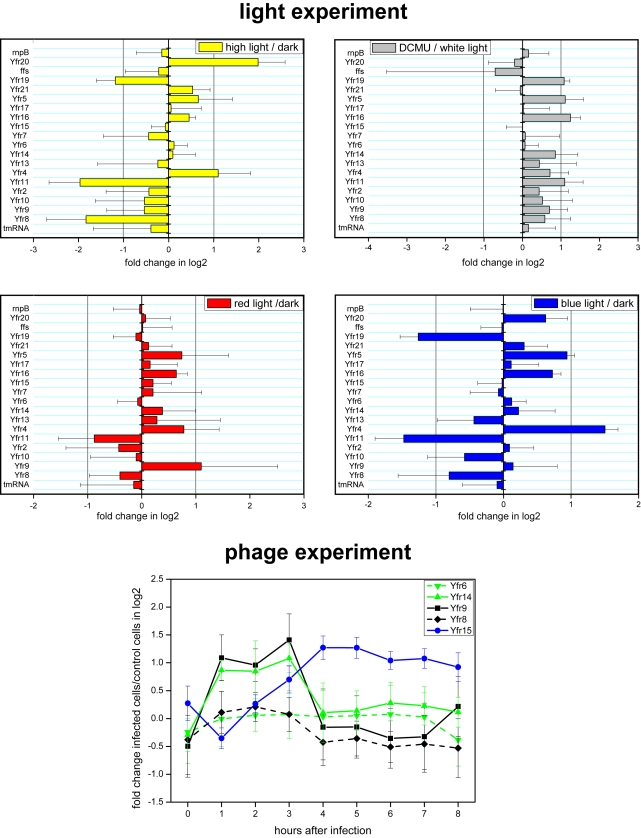
Regulation of ncRNAs under light and phage stress (no changes were found under P-stress). Fold changes (log2) of triplicate experiments are shown. For the light experiment grey vertical lines indicate the border of ≥2-fold change after 45 min of light treatment. Only ncRNAs with significant differential expression and their oppositely located ncRNA pair (when present) are illustrated for the phage experiment. Standard errors derive from experimental repetitions of all probes that gave a signal above threshold.

**Figure 5:**
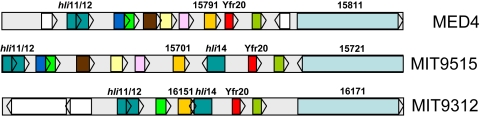
Genomic location of Yfr20 (red boxes) in three high-light adapted *Prochlorococcus* strains. Homologous genes are indicated by the same colors in the different strains and those without homology in this genomic region are shown in white. Gene designations are given according to published genome sequences for MED4 (BX548174.1), MIT9312 (CP000111.1) and MIT9515 (CP000552.1).

Since the expression of many regulatory RNAs is coupled to the process they help regulate [12],[44],[45], we explored the differential expression of the ncRNAs we identified as a function of different environmental stresses. The expression levels of several ncRNAs were influenced by light and phage induced stress, but not by phosphorus stress. Two ncRNAs – Yfr19 and Yfr11 were more than twofold downregulated after transfer from darkness to high white light, normal white light or blue light, but were upregulated when DCMU (an inhibitor of the photosynthetic electron transport chain) was added to cells grown in normal white light conditions (Figure 4, Table S1). Expression of Yfr16, the homolog of Yfr11, followed the same trends, but was less pronounced than for Yfr11. Both the reduced transcript levels during light exposure and the increased amount upon DCMU treatment indicate a link between the redox status of the photosynthetic electron transport chain and these three ncRNAs. High light induced differential expression in the largest number of ncRNAs, and of the highest magnitude, as has been observed for the response of protein-coding genes [38]. However, only a single ncRNA, Yfr20, was upregulated when cells were transferred from darkness to high light (Figure 4, Table S1), whereas all other ncRNAs responsive to light stress decreased in their transcript levels. Yfr20 accumulates in high absolute amounts (Figure 2). According to 5′RACE, the major accumulating transcript of 89 nt results from a specific initiation of transcription at position 1336435 (accession number BX548174.1). In addition 5′RACE results show that Yfr20 is transcribed together with the upstream located ORF PMED4_15791 as a dicistronic element. PMED4_15791 showed constitutive expression. Thus, the light-dependent expression of Yfr20 is under control of its own promoter. The dicistronic gene arrangement with an upstream located ORF is split in *Prochlorococcus* strains MIT9515 and MIT9312 and contains an additional *hli* gene in between the ncRNA and the ORF homologous to PMED4_15791 (Figure 5) providing further evidence for a possible light-regulatory function of Yfr20. Intriguingly, Yfr20 is the only ncRNA encoded in genomic island 5. This island has been characterized as a “phosphorus” island in MED4 since nine genes (nearly all of unknown function) responded when MED4 cells were starved for phosphorus [40]. However, high light stress caused an additional 15 genes to respond in genomic island 5 [11], among them *hli11* and *hli12*, which are located at a distance of less than 2 kb from *yfr20* (Figure 5). Although *hli* (high light inducible) proteins can be factors in other stress responses as well, their mode of regulation here indeed suggests that this island plays a role not only in the adaptation to phosphorus starvation but also to stress caused by high light.

Two distinct stress responses of the cell in response to phage infection have been identified. Lindell and co-workers [39] observed an overall reduction in expression of host genes as the major response to phage infection. However, 41 protein-coding host genes were upregulated in the initial or the mid-to-late phases of phage infection. It is hypothesized that genes belonging to group 1 (the first wave of upregulation) constitute a direct defence to phage infection whereas group 2 genes (the second wave) may be induced by the phage [39]. Two ncRNAs – Yfr9 and Yfr14 - were upregulated in the initial phase of infection (from 1–3 hours after infection, corresponding to group 1 upregulated protein-coding genes [39], Figure 4, Table S1). Interestingly, both ncRNAs have an antisense-located ncRNA – Yfr8 and Yfr6 – that are constitutively expressed during that time (Figure 4, Table S1). The two pairs of overlapping ncRNAs are characterized in more detail below (see section on overlapping ncRNAs). An additional ncRNA – Yfr15 - was upregulated during the mid to late phases of infection (from 3 to 8 hours corresponding to group 2 upregulated protein-coding genes [39], Figure 4, Table S1). Yfr15 is located in genomic island 2 in the vicinity of PMED4_07441 (PMM0686), the most highly upregulated host mRNA during phage infection, although the two genes are located on opposite strands. Also PMED4_07401 (PMM0684) and PMED4_07421 (PMM0685), two further genes that belong to group 2 phage-induced host genes, are located nearby in genomic island 2. This region and Yfr15 may therefore be of prime importance for phage-host interactions.

We did not detect a single ncRNA that was significantly differentially expressed under phosphorus limitation. This was very surprising, in light of the 34 protein encoding-genes that are differentially expressed under P-stress in MED4 [40], and because in *Escherichia coli* the existence of such ncRNAs was hypothesized based on the observation of Hfq-dependent regulation of *rpoS* in response to this stress [46].

### An Ultraconserved Sequence Motif is Present in Three ncRNAs

**Figure 6:**
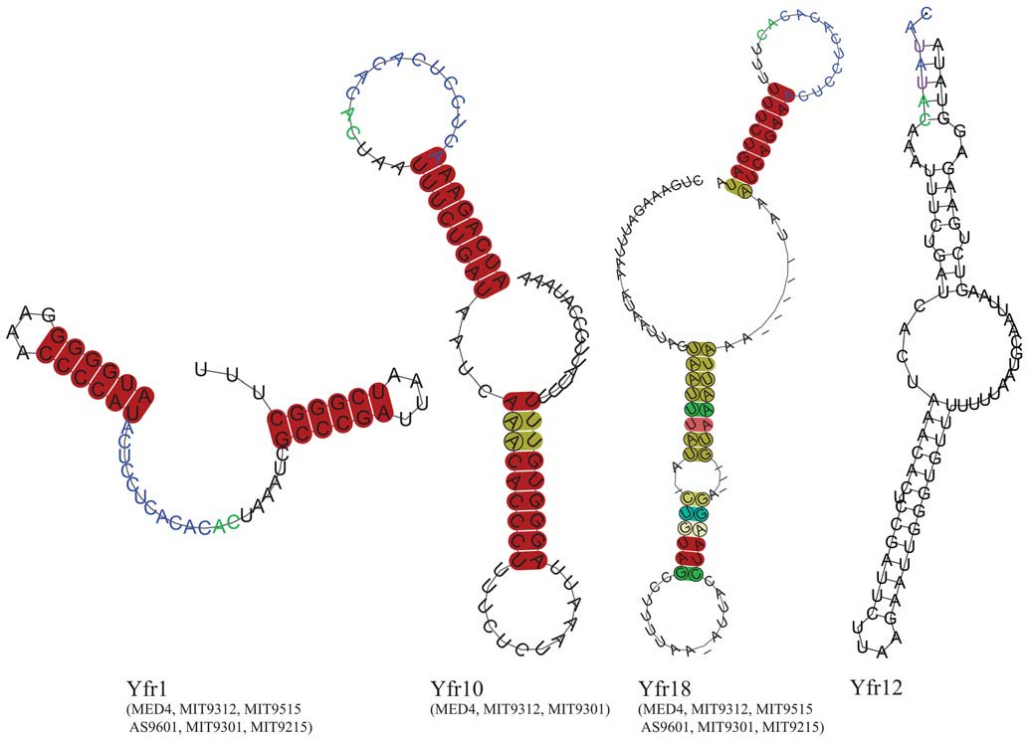
A highly conserved RNA motif. The conserved motif (5′-ACUCCUCACAC-3′) is highlighted in blue and the *Prochlorococcus*–specific extension of the motif is indicated by green letters. The two base mutations in Yfr12 are colored in purple. The color code for shaded bases is as follows: red, all sequences have the same two nucleotides; ochre, two types of base pairs occur; green, three types of base pairs occur; turquoise, four types of base pairs occur. The saturation decreases with the number of sequences unable to form a base pair at this position. Circles around letters indicate compensatory mutations. Consensus structures for Yfr1, Yfr10, Yfr18 were predicted with RNAlishapes [63] and drawn with RNAplot [64]. Homologous ncRNAs found in other *Prochlorococcus* strains that were used for structure predictions are listed in parentheses. The structure of Yfr12 was folded using RNAshapes and relies on the single sequence of Yfr12 from MED4.

The ncRNA Yfr10 contains the conserved unadecanucleotide motif 5′-ACUCCUCACAC-3′ (Figure 6). This motif occurs 3 times in the MED4 genome sequence, which is more frequent than expected by chance: One would expect approximately 0.5 instances of a specific 11 nt motif in a 2 MB genome at equal base distribution. The second occurrence has already been described as belonging to another ncRNA in MED4, Yfr1 [27], which is also found throughout the cyanobacterial radiation.[27]. Using Northern analysis and 5′RACE, we showed that also the third copy of this motif is expressed, revealing another ncRNA - Yfr18 (Figure 2, Table 1). This one was not detected from our microarray analyses nor the comparative genomics approach [25].

If two base transitions are allowed there is even a fourth member of this ‘unadecanucleotide-containing’ class of ncRNAs in MED4. This ncRNA - Yfr12 - was identified by sequence similarity to Yfr10 and was verified as an ncRNA as described above (Figure 2, Table 1). Yfr12 appears to be a mutated variant of the other three, since the processed 5′ end of the major accumulating RNA species was mapped to the middle of the unadecanucleotide, and two mutations change the sequence at the 3′ end of the motif from CACACAC to CAUAUAC (Figure 6). Furthermore, the motif can be extended in all 4 ncRNAs by another AC dinucleotide (Figure 6) in comparison to the published cyanobacterial consensus [27], probably a peculiarity of these RNAs in *Prochlorococcus*.

The functions of Yfr10, Yfr12 and Yfr18 in MED4 remain unknown at present. However, a hint about their potential function may be found from their genome context and the fact that the vast majority of functional interactions between ncRNAs and their targets is exerted through base pairing. The genes for Yfr10, Yfr12 and Yfr18 are each directly adjacent to those for ncRNAs Yfr2, Yfr4 and Yfr5 respectively (Table 1), the 5′ ends of which may basepair to the 13-nucleotide consensus of Yfr1, Yfr10, Yfr12 and Yfr18 if a single bulging C and one mismatch is allowed (i.e. 5′-aCUCCUcACACAC-3′ pairs with 5′-GUGUGUAGGAG-3′). Moreover, one may note that also the two C – to - U transitions in Yfr12 are compatible with this suggested base pairing, and that secondary structure predictions suggest that the conserved motifs in Yfr1, Yfr10 and Yfr18 are exposed as single stranded elements in an otherwise folded region (Figure 6). The same is true for the complementary motif in Yfr2–Yfr5, making physical interactions very likely. Evidence for two regulatory RNAs acting upon each other has recently been reported for the first time for GlmY and GlmZ of *Escherichia coli*, and cascades of hierarchically acting regulatory RNAs have been hypothesized for other bacteria as well [43]. The ncRNAs described in this section are candidates for such interactions in *Prochlorococcus*.

### Two Overlapping Pairs of ncRNAs

**Figure 7:**
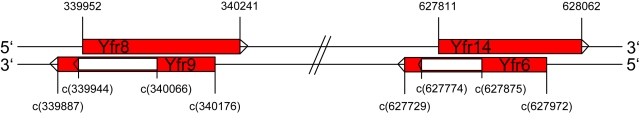
Gene arrangement of ncRNA pairs Yfr8/Yfr9 and Yfr6/Yfr14. Potential peptide-coding open reading frames within the sequence of the RNAs are shown as white boxes. Positions in the genome are indicated by numbers for the forward and reverse (c) strand.

The difference between *trans*- and *cis*-encoded ncRNAs and asRNAs is frequently considered fuzzy since both act through base complementarity. However, depending on the length of the overlap, interactions between transcripts from the forward and the reverse DNA strand can be very strong due to the extended perfect sequence complementarity. asRNAs may act as the antidote in toxin-antitoxin systems [47],[48] or in gene regulation [30],[31] as has been reported for some bacteria. We detected two regions with probable sense/antisense pairing between ncRNAs. One of these regions contains Yfr6 with Yfr14 on the opposite strand (Figure 7, Table 1). The second region is located in genomic island 1 containing Yfr8 and Yfr9, each of which are 290 nt in size (Figures 2 and 7, Table 1). One of the ncRNAs in both pairs is upregulated during phage infection (Figure 4) and both ncRNA pairs contain a potential peptide-coding open reading frame within the sequence of one of the RNAs (Figure 7). The peptide sequence associated with Yfr6 is 33 amino acids long and is highly conserved and widely distributed among high light- and low light *Prochlorococcus* strains. The potential 44 amino acids peptide-coding frame within Yfr9 was found in three other *Prochlorococcus* isolates (MIT9515, MIT9301, AS9601), but has been lost in *Prochlorococcus* strains MIT9312 and MIT9215 due to a frame shift. Homologues of Yfr6 and Yfr9, respectively, have high sequence conservation over their complete ncRNA genes – including the upstream and downstream regions of the potential peptides.

Yfr6 and Yfr9 resemble RNAIII from *Staphylococcus aureus* being both relatively long and consisting of a small peptide-coding unit as well as a regulatory RNA. RNAIII is a 510 nt long riboregulator from which the 26 amino acid δ-hemolysin peptide is also translated [49]. Another bifunctional ncRNA (SgrS) has been described in *Escherichia coli* that contains a conserved ORF (SgrT) in the 5′ region of SgrS, both of which promote recovery from glucose stress in mechanistically distinct fashions [50]. Moreover, secondary structure predictions of Yfr6 [25] and of Yfr14, Yfr8, and Yfr9 (Figures S2 and S3) support the potential role of these transcripts as functional RNAs as they contain many G - C base pairings and compensatory mutations, which conserve the structure rather than the sequence – a feature of many ncRNAs. On the other hand, highly structured transcript regions are also found in certain mRNAs where they serve as platforms for sophisticated ncRNA-mediated translational control. In the case of the *Escherichia coli tisB* mRNA, for example, this transcript encodes a peptide as short as 29 amino acids towards its 3′ end, yet there is no evidence that *tisB* would act as a riboregulator [17],[51].

The fact that Yfr6 and Yfr9 overlap with other ncRNAs (Yfr14 and Yfr8 respectively), suggests that this could be a toxin-antitoxin system – i.e. pairs of genes that code for a stable toxin and an unstable antitoxin. These are well-characterized in other bacteria, where the toxin is usually a toxic peptide that is neutralized or whose synthesis is prevented by the action of the product of the second gene, the antitoxin, which is either protein or RNA. Toxin-antitoxin systems such as the *hok/sok* system of *Escherichia coli* can serve as a natural genetic selection system to ensure presence of a plasmid [52]. Alternatively, chromosomally encoded toxin-antitoxin systems can be beneficial to cell survival under unfavorable growth conditions, sometimes in very sophisticated ways, for instance by transiently curtailing the consumption of nutrients during starvation or by temporarily inhibiting growth and thereby evading the killing effects of certain antibiotics [53]. Systematic searches for toxin-antitoxin systems have revealed a high abundance in free-living prokaryotes [54] but none of the seven known toxin-antitoxin families could be identified in *Prochlorococcus*. There is a growing number of examples of chromosomal toxin-antitoxin systems that use a *cis*-encoded asRNA as an antitoxin. Our data suggest that Yfr6/Yfr14 and Yfr8/Yfr9 may be potential candidates for toxin-antitoxin systems in *Prochlorococcus* MED4.

### A High Number of *cis*-Encoded Antisense RNAs

Little attention has been given to chromosomally *cis*-encoded asRNAs until recently, and only a few have been described for cyanobacteria [29]–[31]. Surprisingly, we detected 24 asRNAs in our analyses, which vary between 100 to 600 nt in size (Table S2). Some are differentially expressed under different light conditions and under phage infection (Table S3).

High light treatment caused one asRNA to be upregulated and one to be downregulated (Table S3). asRNA asMED4_15721 was upregulated twofold when cells were transferred from darkness to high light (Table S3). This behavior is similar to that of Yfr20 (see above), and like Yfr20, this asRNA is located in genomic island 5, lending additional support for a function of this island in light stress adaptation. Notably, five of the 24 asRNAs are complementary to mRNAs that code for photosystem I subunits (*psaB* and *psaC*) or for photosystem II subunits (*psbB* and *psbO*, *psbX*), respectively. The concentrations of asRNAs of photosystem II genes did not change when cultures were shifted from darkness to different light quantities and qualities, whereas transcript levels of their target mRNA decreased slightly (Table S3). In contrast, levels of the photosystem I asRNAs asMED4_17331 (antisense of *psaB*) and asMED4_18171 (antisense of *psaC*) decreased when cells were shifted from darkness to light, following the same trend as their mRNA counterparts (Table S3). Surprisingly, however, the latter asRNAs decreased in amount when transferred from darkness to medium white light whereas their target mRNAs did not (Table S3), which might indicate a light-dosage specific regulation of these asRNAs.

We also found asRNAs that are differentially expressed in cells infected by phage. asMED4_04601 is upregulated during the initial stages of phage infection, whereas its target mRNA (PMED4_04601) is constitutively expressed throughout the infection process. Interestingly, PMED4_04601 shows 67% amino acid identity to the central region of the potential Yfr6 peptide. In the case of PMED4_07401 (PMM0684) both the respective asRNA (asMED4_07401) and its target mRNA are upregulated from mid-to-late phase of phage infection.

The number of 24 asRNAs detected in our analyses appears high, especially as the microarrays used for this study did not contain probes for antisense regions of protein coding genes (see methods). Therefore we could only detect those asRNAs found in intergenic regions whose corresponding ORF was not originally annotated and asRNAs located in 5′ and 3′ UTRs. However, our mapping results revealed that asRNAs located opposite of 5′/3′ UTRs frequently overlap major parts of the adjacent coding sequences. While it is not possible to infer the functions of these asRNAs in *Prochlorococus*, in the cyanobacterium *Synechocystis* PCC6803 the asRNA IsrR occurs in higher quantities than the *cis*-encoded mRNA *isiA* under normal growth conditions, leading to degradation of RNA duplexes by RNase III [30]. Under stress conditions the expression of the mRNA is increased leading to free mRNA molecules that can be translated. This mode of action is highly unlikely in the case of the PMED4_07401 mRNA/asRNA pair, because they are co-upregulated during phage infection, pointing towards a protective rather than a degradative role. Interestingly, RNAse E is also among the genes upregulated during phage infection. It is hypothesized that ribonuclease activity could be utilized by the phage to degrade host RNA to generate nucleotides for phage replication [39].

### Other Overlapping Transcripts

In addition to non-protein coding asRNAs, we found several pairs of transcripts that are transcribed from complementary strands and that potentially code for proteins. These complementary transcripts overlap entirely with each other or with a major part of their 5′ or 3′ UTR. We confirmed these overlapping regions experimentally and found that they span between 74 nt to 333 nt at least (Table S2). Eight out of twelve of these overlapping regions are found in the same position in other *Prochlorococcus* genomes, whereas the other 4 are found in different regions of the genomes. At this point it is not clear whether the overlaps between these protein-encoding transcripts would interfere with their transcription, transcript accumulation or translation. We found evidence for both scenarios. Whereas PMED4_14671 (located in the opposite 3′UTR region of PMED4_14661) is upregulated 14 fold when light intensity is increased, PMED4_14661 (PMM1300) remains at basal transcript levels. Contrary to the above, PMED4_11211 (PMM0997) and PMED4_11201 (located in the opposite 3′UTR region of PMED4_11211) are inversely regulated under different light conditions and DCMU treatment (Table S4) indicative of either interference during transcription, or coupled degradation, as observed for the asRNA and mRNA IsrR/*isiA* in the cyanobacterium *Synechocystis* PCC6803 [30].

In cases where only 3′-UTRs overlap this might not be of relevance because the transcriptional machinery should not be constricted. However, most of the transcripts we found overlap the 5′-UTRs and/or complete protein-coding regions and therefore are highly likely to be of regulatory relevance.

### Identification of New ORFs

It is difficult to identify short genes in bacterial genomes using annotation algorithms, because the number of possible reading frames increases the shorter the search window becomes. Therefore, many of the widely used annotation programs (e.g. GLIMMER, GeneMark and CRITICA) constrain the minimum length of an ORF and thus a considerable number of small ORFs remain unannotated. In a recent study of the *Prochlorococcus* pan genome, for example, hypothetical ORFs shorter than 50 amino acids were excluded unless they were found in more than one genome [9].

Our microarray analyses lead to the observation of 113 new ORFs (not including asRNAs with potential protein coding sequences; Table S2) ranging between 33 to 130 amino acids in size that were not annotated in the first published *Prochlorococcus* MED4 genome version (accession number BX548174). The new annotation of Kettler et al. [9] (accession number BX548174.1, for new ORF IDs refer to: www.microbesonline.org) also found 89 of the new ORFs. BlastP searches against the non-redundant NCBI database revealed that 10 of the 24 remaining ORFs have an annotated counterpart in at least one other genome whereas 14 ORFs represent short proteins that have not been detected thus far (Table S5). Using TblastN, all of the additional 14 novel ORFs were found in other genomes, even though they had gone undetected by computational annotation tools (Table S6).

### Phage-Related Open Reading Frames

Amongst the newly discovered protein-coding genes are three ORFs that have homologues in cyanophage genomes. PMED4_16122, which is located in genomic island 5 in MED4, is homologous to PSSM4_095 in the MED4-infecting cyanophage PSSM4 [55], suggesting gene transfer between a PSSM4-like phage and this island, in particular since no other homologues were found in the nr database. PMED4_15491 is in vicinity of genomic island 5 in the host genome and has one homolog in each of two cyanophage genomes – P-SSM4 and P-SSM2 (PSSM4_181 and PSSM2_278). According to TblastN results this ORF is present in numerous *Prochlorococcus* genomes but not in other cyanobacteria. The third newly discovered ORF with a homolog in a cyanophage is PMED4_10681, which is found in the genome of cyanophage P-SSM2. Unlike PMED4_16122, however, this ORF is not in a genomic island in the host genome, and furthermore, is widely distributed over the cyanobacterial radiation with homologues in all *Prochlorococcus* strains, *Synechococcus elongatus* strains PCC 6301 and PCC 7942, *Fremyella diplosiphon*, *Nostoc* sp. PCC 7120, *Anabaena variabilis* and *Synechocystis* sp. PCC 6803 (Table S6). The broad distribution of PMED4_10681 suggests that it plays an important function in cyanobacteria, and emphasizes the importance of better annotation of small ORFs.

### Conclusions

Here we have described 14 novel ncRNAs, which increases the total number of ncRNAs in this organism to 24 (including Yfr1-7, ffs, tmRNA, RNase P RNA). One sixth of the 24 ncRNAs (Yfr1, Yfr3, Yfr12 and Yfr18) were undetectable from microarray analyses under the conditions tested. Therefore it is likely that even more ncRNAs are present in *Prochlorococcus* MED4. The proportion of ncRNAs in the *Prochlorococcus* MED4 genome is comparable with those found in enterobacteria like *Escherichia coli*, i.e. 1–2% of the genes encode ncRNAs. In comparison, the 6 identified protein regulators in *Prochlorococcus* [10] is a small number relative to the 32 two-component response regulators present in *Escherichia coli* [56]. This suggests that regulation of gene expression through ncRNAs plays an important role in *Prochlorococcus*' response to environmental cues. The relatively high number of ncRNAs is intriguing as it may represent a mode of adaptation to the extremely low nutrient conditions of the open oceans. Regulation by ncRNAs may require fewer resources than would be required for the synthesis of protein regulators. Furthermore, in the course of genome reduction there might have been a positive selection pressure for keeping small regulators, e.g. ncRNAs rather than large protein regulators. How ncRNAs function in *Prochlorococcus* is at present unclear. The absence of Hfq in MED4 suggests that the ncRNAs found in this strain represent a core-set of ncRNAs that function without the support of a chaperone, or with a novel chaperone yet to be identified.

The genomic islands of *Prochlorococcus* are disproportionately connected to ecological functions in this group of cyanobacteria [57],[11]. Here we have shown that approximately half of the *Prochlorococcus* ncRNAs are located in genomic islands suggesting that the function of these molecules is relevant for determining the relative fitness of ecotypes within *Prochlorococcus*. This is analogous to the accumulation of genes coding for ncRNA in pathogenicity islands in *Staphylococcus aureus* [58] and *Salmonella typhimurium* [59] as well as in genomic islands of *Sinorhizobium meliloti* [60], and suggests that this phenomenon could be wide-spread for finely tuned specialization within microbial groups.

## Materials and Methods

### Extraction of Microarray Expression Signals

Three independent microarray experiments investigating global changes of gene expression under different light quantities and qualities (light experiment, [38]), under phage infection (phage experiment, [39]) and under phosphorus starvation (phosphorus experiment, [40]) were analyzed. The custom Affymetrix high-density array MED4-9313 that was used features 25-base oligomers identical to the target sequence that are spread over the complete genome comprising all gene coding regions as well as all intergenic regions on both forward and reverse strands with a coverage of every 45 bases in intergenic regions, a special feature that offers the detection of unknown transcripts. The Affymetrix array also contains probes for another *Prochlorococcus* genome (MIT9313) and two cyanophage genomes P-SSP7 and P-SSM4, whose average signal intensities were used to calculate threshold expression signals. For each set of experiments the threshold value used was re-evaluated to ensure high specificity of candidate probes. Specifically, we extracted probes with an expression signal of ≥200 in 18 of 21 arrays from the light experiment. Because of different experimental designs and thus resultant variations in overall expression signals, the threshold filter was adapted for the phage and phosphorus experiment extracting probes with expression signals ≥100 in 4 of 14 or 4 of 10 time points respectively (corresponding to the average of biological triplicates), respectively, with a 2-fold change in at least one time point between control and stress condition. The distribution of probe intensities was adjusted by quantile normalization across different arrays within the same experiment. This procedure minimized array-specific effects and allowed us to determine fold changes of single probes targeting ncRNAs, asRNAs and overlapping transcripts. Rather strict criteria for transcript identification were chosen to ensure a high true positive rate for transcript detection. To minimize the number of 5′ and 3′UTRs detected, probes within 100 nt of the adjacent gene in the same orientation were excluded. Remaining probes were grouped in transcriptional units and further characterized to categories: ORF, asRNA, ncRNA, 5′/3′UTR, pseudogenes and operon elements. The grouping and characterization is based on the localization in the genome and on BLAST searches against 11 *Prochlorococcus* genomes (http://www.ncbi.nlm.nih.gov/genomes/MICROBES/microbial_taxtree.html) to identify conserved regions. Genes classified as ORFs encode for a peptide sequence with a start and stop codon without a frame shift and were present in at least two genomes. All PMED_xxxxx ORF notations (including new ORFs) follow that of Kettler et al. [9] and are available at www.microbesonline.org. ncRNAs and asRNAs were defined as genes without peptide-coding potential localized in intergenic regions and opposite protein-coding genes, respectively. In two special cases ncRNAs with a regulatory RNA component as well as a peptide-encoded component were allowed. For detailed information about grouping see Table S2.

### Culture Conditions

*Prochlorococcus* MED4 was grown at 21°C in AMP1 medium [61] under 30 µmol quanta m^−2^ s^−1^ continuous white cool light. Culture conditions for microarray experiments are provided elsewhere [39],[40],[38].

### 5′- and 3′-RACE

Total RNA was isolated as previously described [38] with the following modifications. Cells were harvested by centrifugation at 10,000×g for 10 min at 20°C. The pellet was resuspended in RNA resuspension buffer (10 mM sodium acetate [pH 5.2], 200 mM sucrose, 5 mM EDTA), snap frozen in liquid nitrogen and subsequently stored at −80°C. Total nucleic acids were DNase-treated with Turbo DNA-free (1 U/8 µg RNA, Ambion, USA) for 15 min at 37°C. RNA was precipitated with 1/10 volume 3 M sodium acetate (pH 5.2) and 3 volumes ethanol by centrifugation at 13,000×g for 30 min at 4°C and subsequently resuspended in water. Transcriptional start sites were determined by 5′-RACE following the method of Bensing et al. [62]. Briefly, RNA was treated with tobacco acid pyrophosphorylase (1 U/1 µg RNA; Epicentre, USA) for 1 h at 37°C followed by phenol/chloroform extraction and ethanol precipitation. A synthetic RNA oligonucleotide (0.5 µl oligonucleotide [10 µM]/ 4 µg RNA; AUA UGC GCG AAU UCC UGU AGA ACG AAC ACU AGA AGA AA, Invitrogen, Germany) was ligated to RNA using T4 RNA ligase (3 U/1 µg RNA; Fermentas, Germany) for 1 h at 37°C followed by phenol/chloroform extraction and ethanol precipitation. Three control reactions were performed: i) omitting tobacco acid pyrophosphorylase, ii) omitting tobacco acid pyrophosphorylase and RNA oligonucleotide and iii) dephosphorylating RNA prior to ligation with calf intestine alkaline phosphatase (0.1 U/1 µg RNA; Fermentas, Germany) at 37°C for 1 h, followed by phenol/chloroform extraction and ethanol precipitation. For reverse transcription 250 ng linked RNA per gene was incubated with 0.8 U of the Omniscript reverse transcriptase (Qiagen, Germany) in the provided reaction buffer containing 0.08 µM gene specific primer and 1 mM dNTPs. Incubation was carried out at 42°C for 2 h with a final inactivation step at 95°C for 5 min. All reactions were performed in the presence of 40 U Ribolock RNase Inhibitor (Fermentas, Germany). cDNA was amplified by PCR using a gene-specific primer (0.2 µM) and an RNA oligonucleotide-specific primer (0.2 µM) with following the cycling conditions: 93°C/3 min; 35 cycles of 93°C/30 s; 50°C/30 or 55°C/30 or 60°C/30 s, 72°C/45 s; 72°C/5 min in GoTaq reaction buffer containing 1 U GoTaq polymerase (Promega, Germany), 0.2 mM dNTPs and 3.5 mM MgCl_2_. A complete list with all primers used is provided in Table S7. Amplified PCR fragments were gel-excised and purified on Nucleospin columns (Macherey & Nagel, Germany) and then cloned into plasmid pGEMT (Promega, Germany). After transformation into *E. coli* XL1-Blue, plasmid inserts were amplified by colony PCR, purified on Nucleospin columns (Macherey & Nagel, Germany) and sequenced using an ABI 3130XL automatic DNA sequencer (Applied Biosystems, USA). To determine the 3′ end of RNAs, 3′RACE was performed following the method described previously [17]. Briefly, RNA was treated as described above followed by a dephosphorylation with calf intestine alkaline phosphatase (0.2 U/1 µg RNA; Fermentas, Germany) at 37°C for 1 h and a subsequent phenol/chloroform extraction and ethanol precipitation. RNA 3′ ends were linked to a 3′ end blocked RNA oligonucleotide (0.5 µl oligonucleotide [10 µM]/4 µg RNA, pAAG AUG AAU GCA ACA CUU CUG UAC GAC UAG AGC AC, Metabion, Germany) using 0.8 U/1 µg RNA T4 RNA Ligase (Fermentas, Germany) followed by phenol/chloroform extraction and ethanol precipitation. Reverse transcription was performed as described above with the following modifications: 0.2 µM 3′ RNA oligonucleotide-specific primer and 2.5 mM dNTPs. Subsequent PCR, cloning and sequencing was performed as described above. Determined 5′ and 3′ ends are given in Tables S2 and S8.

### Northern Analysis

RNA samples (50 µg) were denatured for 5 min at 65°C in loading buffer (Fermentas, Germany), separated on 10% urea-polyacrylamide gels for 16 h at 100 V and transferred to Hybond-N nylon membranes (Amersham, Germany) by electroblotting for 1 h at 400 mA. The membranes were hybridized with specific [γ -^32^P]ATP end-labelled oligonucleotides or [α-^32^P]UTP-incorporated transcripts. Hybridization in 50% deionized formamide, 7% SDS, 250 mM NaCl and 120 mM Na(PO4) pH 7.2 was performed over night at 42°C or at 62°C with labelled oligonucleotide probes or labelled transcript probes, respectively. The membranes were washed in 2×SSC (3 M NaCl, 0.3 M sodium citrate, pH 7.0) [55], 1% SDS for 10 minutes; 1×SSC, 0.5% SDS for 10 min; and briefly in 0.1×SSC, 0.1% SDS. All wash steps were performed 5°C below hybridization temperature. Signals were detected and analyzed on a Personal Molecular Imager FX system with Quantity One software (BIO-RAD, Germany).

### Oligonucleotide End Labelling

Gene-specific oligonucleotides were labelled with [γ-^32^P]ATP by the exchange reaction of T4 polynucleotide kinase (Fermentas, Germany) using 0.5 U of enzyme, 1.25 µM oligonucleotide, 15 µCi [γ-^32^P]ATP in reaction buffer A for 30 min at 37°C followed by inactivation for 5 min at 95°C.

### In Vitro Transcription

The MAXIscript Kit (Ambion, USA) was used for transcription of probes for use in Northern analyses containing 100 ng PCR-generated DNA template, 500 µM each of ATP, CTP, GTP, 20 µM UTP, 50 µCi [α-^32^P]UTP, 1 µl T7 enzyme mix in reaction buffer amended with SUPERase In RNase inhibitor (Ambion, USA). Transcription was carried out at 37°C for 10 min. Thereafter, the reactions were treated with 2 U of Turbo DNase-free (Ambion, USA) at 37°C for 15 min. The enzyme was heat inactivated for 10 min in the presence of 23 mM EDTA.

## Supporting Information

Figure S1 Structures of Yfr11 and Yfr16. The structures were predicted in RNAlishapes [61] and were drawn with RNAViz [64]. Conserved bases are shaded in grey.
(1.41 MB EPS)

Figure S2 Consensus structures of Yfr6 and Yfr14. The structures were predicted with RNAlishapes [61] and drawn with RNAplot [62] and are based on sequence alignments including MED4, MIT9312, MIT9515, MIT9301, AS9601, NATL1A and SS120 (in parenthesis). The color code is the same as for Figure 6.
(1.43 MB EPS)

Figure S3 Consensus structures of Yfr8 and Yfr9. The structures were predicted with RNAlishapes [61] and drawn with RNAplot [62] and are based on sequence alignments including MED4, MIT9312, MIT9515, MIT9301, MIT9215 and AS9601 (in parenthesis). The color code is the same as for Figure 6.
(2.66 MB EPS)

Table S1 Differential expression of ncRNAs during light, phage and phosphorus stress. Microarray ratios are average values ± standard errors of all probes (with expression values above threshold) targeting the respective ncRNA for triplicate biological repeats.
(0.07 MB XLS)

Table S2 Transcripts detected from microarray expression analysis classified as new ncRNAs, asRNAs, small ORFs, pseudogenes and UTR regions of adjacent genes. Capital letters and underlined letters in columns “Sequence 5′end” and “Sequence 3′end” indicate mapped ends and TATA boxes, respectively. Transcript detection included microarray analysis (M), 5′ and 3′ RACE (5′R and 3′R), and Northern hybridization (N). For ORFs that were verified by 5′RACE numbers given in brackets correspond to coordinates of the CDS and numbers without brackets indicate mapped 5′ and 3′ ends.
(0.07 MB XLS)

Table S3 Differential expression of asRNAs and their sense transcripts during light, phage and phosphorus stress. asRNAs were named with the prefix “as” according to the gene ID found at www.microbesonline.org for the respective gene they are overlapping with. Microarray ratios are average values ± standard errors of all probes targeting the respective asRNA and its sense transcript for biological triplicates.
(0.10 MB XLS)

Table S4 Differential expression of overlapping transcripts during light, phage and phosphorus stress. Microarray ratios are average values ± standard errors of all probes targeting the respective transcript for biological triplicates.
(0.06 MB XLS)

Table S5 BlastP results for new protein-coding genes against the non-redundant NCBI database. Blast search criteria were set to a cut-off E-value below 0.05 using default algorithm parameters except for compositional adjustment that was disabled. Blasts were performed in February 2008 against the NCBI non-redundant database.
(0.20 MB XLS)

Table S6 TblastN results for new protein-coding genes against the non-redundant NCBI database. Blast search criteria were set to a cut-off E-value below 0.05 using default algorithm parameters except for compositional adjustment that was disabled. Blasts were performed in February 2008 against the NCBI non-redundant database.
(0.08 MB XLS)

Table S7 List of used oligonucleotides.
(0.04 MB XLS)

Table S8 Mapped 5′ends of ORFs opposite of asRNAs that were not detected by the microarray screen.
(0.02 MB XLS)
