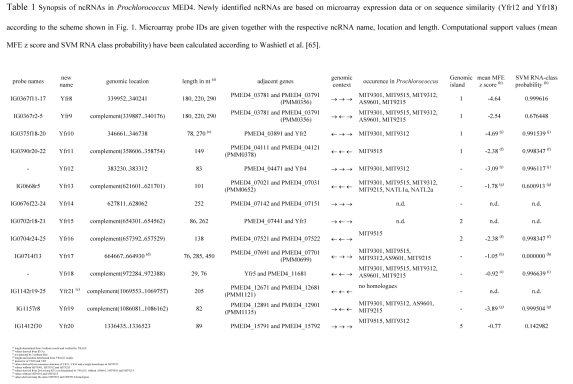# Correction: The Challenge of Regulation in a Minimal Photoautotroph: Non-Coding RNAs in *Prochlorococcus*

**DOI:** 10.1371/annotation/411b74ae-c4ce-43c9-bdd2-60c2bf60e672

**Published:** 2008-11-08

**Authors:** Claudia Steglich, Matthias E. Futschik, Debbie Lindell, Bjoern Voss, Sallie W. Chisholm, Wolfgang R. Hess

Table 1 was not formatted correctly in the published article. The correct version of Table 1 is available here:

**Figure:**